# Mechanistic and Biomarker Studies to Demonstrate Immune Tolerance in Multiple Sclerosis

**DOI:** 10.3389/fimmu.2021.787498

**Published:** 2022-01-05

**Authors:** María José Docampo, Andreas Lutterotti, Mireia Sospedra, Roland Martin

**Affiliations:** Neuroimmunology and Multiple Sclerosis Research Section, Neurology Clinic, University Hospital Zurich & University of Zurich, Zurich, Switzerland

**Keywords:** multiple sclerosis, peripheral tolerance, mechanistic studies, antigen-specificity, autoreactive cell, regulatory T cells, biomarkers, tolerance induction

## Abstract

The induction of specific immunological tolerance represents an important therapeutic goal for multiple sclerosis and other autoimmune diseases. Sound knowledge of the target antigens, the underlying pathomechanisms of the disease and the presumed mechanisms of action of the respective tolerance-inducing approach are essential for successful translation. Furthermore, suitable tools and assays to evaluate the induction of immune tolerance are key aspects for the development of such treatments. However, investigation of the mechanisms of action underlying tolerance induction poses several challenges. The optimization of sensitive, robust methods which allow the assessment of low frequency autoreactive T cells and the long-term reduction or change of their responses, the detection of regulatory cell populations and their immune mediators, as well as the validation of specific biomarkers indicating reduction of inflammation and damage, are needed to develop tolerance-inducing approaches successfully to patients. This short review focuses on how to demonstrate mechanistic proof-of-concept in antigen-specific tolerance-inducing therapies in MS.

## Introduction

Multiple sclerosis (MS) is considered a prototypic organ-specific autoimmune disease that affects the central nervous system (CNS; brain and spinal cord) of young adults and particularly women. In most cases MS begins between 20-40 years of age but may also start in childhood or later in life. There are two main forms with respect to clinical course. Relapsing-remitting MS (RMS) is characterized by bouts of disease activity in different CNS areas that might affect vision, sensation, motor-, bladder-, bowel and sexual function. Initially, the deficits are only transient and often completely recover. RMS is diagnosed based on clinical presentation as well as typical magnetic resonance imaging (MRI) lesions and signs of inflammation in the cerebrospinal fluid (CSF) (1). Pre-stages of RMS are the so-called clinically isolated syndrome (CIS), i.e. a first clinical event with suggestive MRI and CSF findings, or even the accidental discovery of MRI lesions without any prior clinical symptoms, which is referred to as radiologically isolated syndrome (RIS). Prior to the era of effective disease-modifying immunomodulatory treatments, RMS usually evolved into secondary progressive MS (SPMS) after 10-20 years. At this stage, neurological deficits and disability of the patients steadily worsen with or without superimposed relapses, which later completely stop. 80-85% of patients show one of these stages of RIS-CIS-RMS-SPMS. A smaller fraction (10-15%) shows progressive increase of disability from the beginning usually with insidious onset of walking problems. This form is referred to as primary progressive MS (PPMS) and affects women and men equally. Besides these different forms of MS with respect to disease course, there is substantial variation in how quickly neurological deficits develop. Few patients have a benign course (approximately 5%), the majority will develop disabilities over 2-3 decades, if they are not treated, and another, smaller portion (approximately 5-10%) shows rapid disease progression with severe deficits in a few years. In addition to variation in disease course, heterogeneity is also seen with respect to clinical presentation, neuropathological findings, distribution of lesions in the brain and spinal cord and response to treatment.

Although the etiology and pathomechanisms of MS are not yet completely understood, enormous progress has been made during the last 20 years. Genome-wide association studies have characterized the complex genetic trait that confers MS risk with now more than 240 single nucleotide polymorphisms (SNPs) across the genome (2). The vast majority of these and particularly the most important MS risk genes, i.e. the two HLA-DR15 alleles (3), are immune function-related (4). Environmental risk factors include Epstein Barr virus (EBV), low vitamin D3, smoking, obesity during early adolescence (5) and imbalances of gut microbiota (6–8). Differences in the interplay of genetic and environmental risk factors are likely responsible for the heterogeneity of MS with respect to clinical course and involvement of different functional systems of the CNS, imaging findings, pathology and response to treatment.

The development of treatments for MS has been very successful during the last 25 years. More than 20 treatments are now approved including variations in dosing or application forms. They reach from moderately effective injectables and small molecules (IFN-β, glatiramer acetate, teriflunomide) to highly effective biologics such as anti-CD20, anti-VLA4, anti-CD52 and the small molecule cladribine. All act by immunomodulation and/or -suppression, but by different mechanisms (9). The most important effects target autoreactive CD4+ T cells and/or B cells, but to various degrees also innate immune cells. Autologous hematopoietic stem cell transplantation (aHSCT), which is only approved in some countries, is an exception. It primarily acts by completely abrogating the patient’s adaptive immune system and then forming a new one from autologous CD34+ hematopoietic stem cells (10).

In contrast to the above, antigen-specific tolerance induction aims at a subtle readjustment of perturbed immune reactivity, which harms CNS tissue. The currently pursued approaches employ mechanisms of peripheral immune tolerance in the physiological situation, and they are therefore expected to be very safe and not to impair protective immunity against infections and tumors. If immune tolerance-inducing approaches shall successfully enter the clinic and hopefully acquire a firm place in our treatment armamentarium, clinical efficacy, i.e. the reduction of relapses and/or the attenuation of disease progression need to be shown (11). Development of treatments towards approval follows certain standards in MS. Clinical efficacy needs to be documented by two positive phase III trials, which are very costly and usually performed at up to 100 or more sites. For immune tolerance induction, stopping the disease evolution at very early stages, i.e. RIS or CIS, is of particular interest. If robust predictive biomarkers were available, antigen-specific tolerance induction would be even more interesting as a true prophylactic measure to prevent the development of MS. So far, it has, however, been very difficult to overcome the hurdles during the earlier clinical trial stages, i.e. phase IIa and -b.

Below we will outline which immunological mechanisms contribute to MS, how peripheral immune tolerance is generated and maintained, at which aspects of these tolerizing strategies aim, and which factors need to be considered to demonstrate at the mechanistic level whether tolerance induction has been achieved in patients. The challenges of gathering evidence for mechanistic proof-of-concept particularly in early-stage clinical trials will be discussed including which methodologies are currently available.

## Pathomechanisms of MS

Understanding the autoimmune process and target antigens are required for measuring changes after tolerization. Below, we will summarize these, but only briefly mention target antigens in MS, since these has been covered in detail elsewhere recently (11). Also, while it is clear that innate immune cells such as dendritic cells and microglia are involved at different steps of the pathogenesis of MS, we will focus on adaptive immune mechanisms, since these are most relevant for antigen-specific tolerization.

The pathomechanisms of MS involve autoreactive CD4+ T cells with specificity for myelin- and a few other proteins and peptides thereof (4, 12–14), proinflammatory B cells (15) and possibly also autoantibodies (16), but likely also other cell types including CD8+ T cells (17), microglia and other innate immune cells (18). The strong association with a specific HLA-DR haplotype (3), the large body of evidence from experimental autoimmune encephalomyelitis (EAE) studies (19), and also the studies of immune mechanisms in MS patients underscore the central role of autoreactive CD4+ T cells (4, 20). Consistent with the fact that MS only affects the CNS and that demyelination is a key aspect of MS lesions, but also with data from EAE studies, autoreactive CD4+ T cells recognize peptides from several myelin proteins including myelin basic protein (MBP), proteolipid protein (PLP), myelin oligodendrocyte glycoprotein (MOG) and a few others [recently summarized in detail in (11, 21)]. Based on T cell recognition with higher antigen avidity (22), data from humanized transgenic mouse models (23, 24) and from epitope mapping studies, a few immunodominant epitopes of MBP, PLP and MOG appear particularly important (11), but non-myelin antigens including alpha-B crystallin (25), GDP L-fucose synthase (GDPLFS) (13), and RAS guanyl-releasing protein 2 (RASGRP2) (14, 26) should also be considered. Both GDPLFS and RASGRP2 have been discovered by searching for the specificity of CD4+ T cells that were clonally expanded in active MS brain lesions (13, 14). Further, MS patients with intrathecal T cell reactivity against human GDPLFS also recognized homologue bacterial peptides from a gut bacteria that is overrepresented in MS patients, *Akkermansia muciniphila* (13). RASGRP2 is not only expressed by cortical neurons in the brain, but also by proinflammatory B cells that activate autoreactive T cells (14). The antigen-specific T cell response may broaden over time, a phenomenon that is referred to as epitope spreading (27) and means that additional antigen specificities emerge and/or prior ones are lost. Spreading can be intramolecularly, i.e. to a new peptide of the same protein, or intermolecularly, i.e. a peptide from another target protein. Epitope spreading has been examined in detail in EAE (28), but only few studies have addressed it in MS patients (29). In the context of tolerance induction, it implies that not only the antigens contained in the tolerance-inducing approach, but also other candidate targets should be assessed.

With respect to their functional phenotype, autoreactive CD4+ T cells in MS express T helper 1 (Th1; produce IFN-γ), Th1*- (produce IL-17 in addition to IFN-γ) or Th17- (express IL-17) phenotypes and furthermore markers that are important for brain homing such as VLA-4, and the chemokine receptors CXCR3 and CCR6 (30). In our studies of both peripheral blood- and cerebrospinal fluid (CSF)-derived T cell clones in MS, the hierarchy of importance is Th1>Th1*>Th17 cells.

Besides T cell-mediated autoreactivity, autoantibodies have long been considered important in MS pathogenesis. Antibodies that are produced in the CSF as oligoclonal bands (OCBs) are known for more than 70 years and are a diagnostic hallmark in MS, but the pathogenic importance of both OCBs and in general autoantibodies in MS remains controversial (16). However, there is a pathologically defined pattern II MS, in which immunoglobulin and complement factor deposition in the brain (31), the therapeutic responsiveness to plasmapheresis (32), and the recent demonstration of autoreactive Th2 CD4+ T cells (33), all support that antibodies play a role. To our knowledge, no biomarker in the blood and CSF has been identified that allows the identification of pattern II MS patients.

While autoantibody production is probably less important overall in MS, there is no doubt that B cells play an important role. B cell-depleting therapies with anti-CD20 monoclonal antibodies, but also with anti-CD52 and cladribine, which in addition to B cells eliminate other immune cells, are among the most effective therapies for MS (34, 35). The observation that disease activity decreased much earlier after anti-CD20 treatment than expected from removal of antibodies, stimulated the search for additional roles of B cells in MS, including cytokine/chemokine mediated regulation of inflammation and antigen-presentation. During the last years, several studies have shown increased frequencies of B cells that secrete GM-CSF and IL-6 (15) and express other proinflammatory cytokines and chemokine receptors involved in brain homing and interaction with autoreactive T cells (36). Further, proinflammatory memory B cells appear to be involved in presenting antigen to autoreactive T cells, in their activation, and priming for brain homing (14). Peptides derived from the MS-associated HLA-DR15 molecules and upregulation of DR15 itself on the surface of B cells are involved in cross-talk and increased autoproliferation of both B- and autoreactive CD4+ T cells (26), however, it is not clear yet whether the activation occurs first in the B- or T cell. Furthermore, RASGRP2, one of the novel autoantigens is upregulated in proinflammatory B cells in MS and can be cross-recognized by autoreactive T cells that also respond to EBV- and *Akkermansia*-derived peptides (26). In this context it is important to note that EBV, a key environmental risk factor of MS, infects B cells (37), and the risk to develop MS is increased several-fold after symptomatic EBV infection, i.e. infectious mononucleosis (38). EBV infection of B cells and also T cell reactivity to EBV have been implicated in multiple ways both in the peripheral immune compartment and also the CNS (39, 40). Tertiary lymphoid structures in the meninges, which contain B- and T cells, have been linked to cortical lesion formation in MS and also to progressive disease (41, 42). At the latter stage, a compartmentalized chronic immune response in the CNS/meninges is suspected to drive the disease process (43).

In summary, it is clear that autoreactive CD4+ T cells with a Th1- or Th1*-, and, in pattern II MS patients, also Th2 cells as well as proinflammatory B cells play key roles in several steps of the autoimmune pathogenesis of MS. For more detail, the reader is referred to reviews on this topic (4, 44).

## Peripheral Immune Tolerance Mechanisms

The multitude of tolerance mechanisms in humans in both health and disease are incompletely understood. Below, we will mention key aspects that are relevant for characterizing these before and after attempts of tolerance induction.

Immunological tolerance to self-antigens results from both central and peripheral mechanisms. The elimination of strongly self-reactive lymphocytes is controlled by central tolerance mechanisms in the thymus and bone marrow for T and B cells, respectively. During thymic development, T cells which recognize self-antigens with high avidity, undergo negative selection *via* clonal deletion, whereas those recognizing antigens with low avidity are positively selected and constitute the peripheral immune repertoire. T cells bearing TCRs for antigens not expressed in the thymus may, however, not be deleted (45, 46), and low avidity autoreactive T cells responding to myelin antigens can be found in both healthy individuals MS patients (47–50). Such potentially pathogenic cells are controlled by several checkpoints that operate at different stages to avoid the development of autoimmunity.

**Figure 1:**
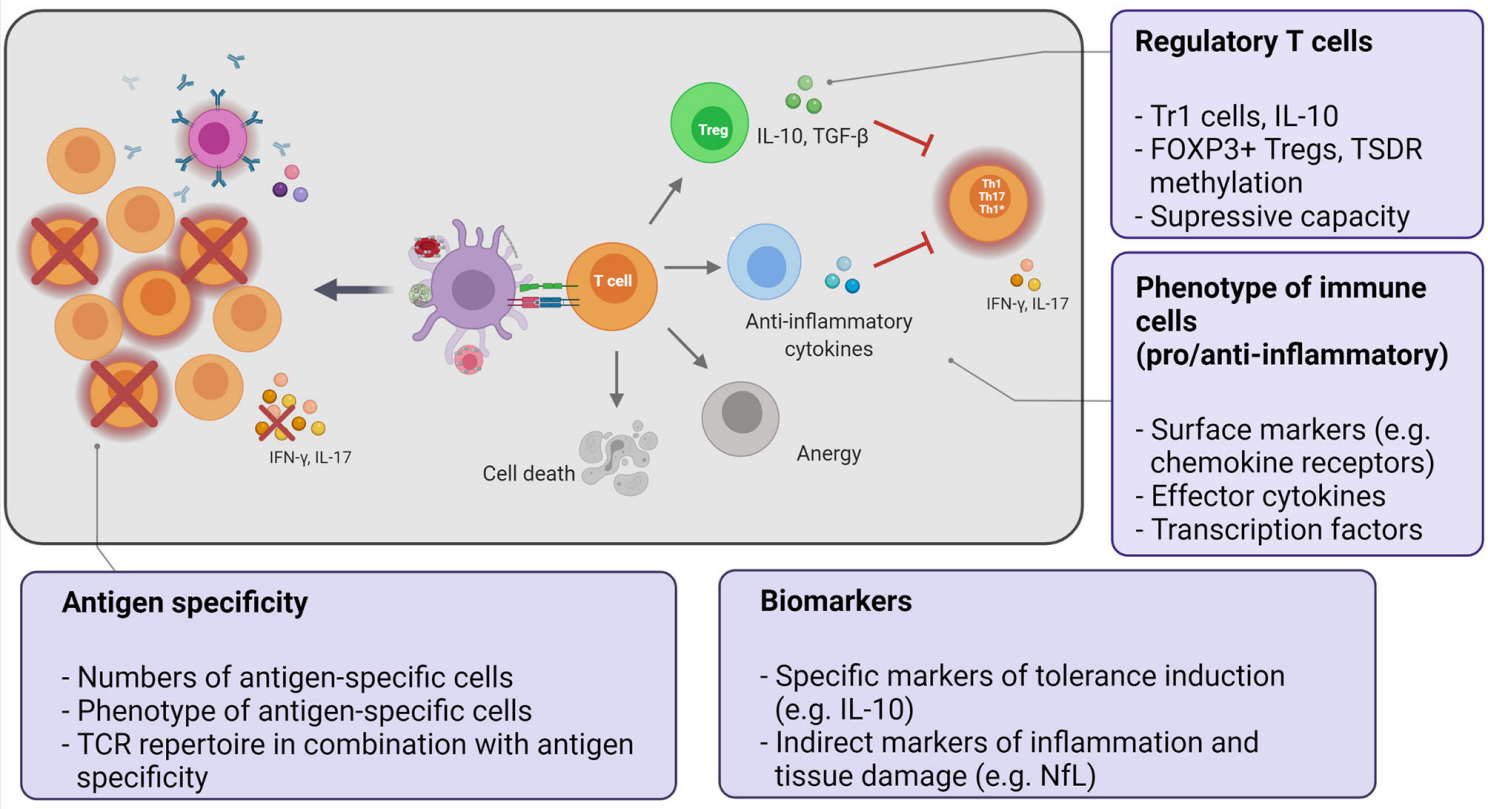
Key aspects of the mechanistic program to monitor immune tolerance. Tolerization strategies employ different approaches to deliver the autoantigen to tolerogenic antigen-presenting cells in order to induce or enhance peripheral tolerance mechanisms. The potential mechanisms of tolerance induction can directly delete or silence autoreactive cells or indirectly suppress them by induction of regulatory T cells or effector T cells producing immunomodulatory cytokines (immune deviation). The final outcome is the reduction of autoreactive, pathogenic effector cells (Th1/Th17/Th1*). Text bubbles summarize the key aspects of the mechanistic studies.

Among peripheral tolerance mechanisms, one is ignorance of self-antigens, either because anatomical barriers limit accessibility (for example the blood-brain-barrier), or because it is present at too low concentrations, or the expression of HLA molecules is limited or absent (51). Although CD4+ MBP-specific T cells in MS primarily derive from the naïve repertoire and show higher antigen avidity (52), they lack adhesion molecules and chemokines receptors that are necessary for organ homing. T cell activation in the absence of costimulatory signals results in a state of unresponsiveness, referred to as anergy. Certain tolerizing approaches induce anergy (53), which can be overcome by IL-2 and therefore is not durable. T cell responsiveness can further be controlled when antigen recognition occurs in the context of a growing family of so-called co-inhibitory molecules including CTLA-4, PD1, TIGIT, BTLA4, LAG-3, TIM-3 (54). Different from anergy, apoptotic deletion of autoreactive cells by activation-induced cell death (AICD), is triggered when already activated cells are newly stimulated with antigen. AICD is mediated by Fas-Fas ligand interaction (55). Additional peripheral cell death checkpoints other than apoptosis also play a role in maintaining tolerance. Further, antigen-induced T cell exhaustion and senescence are non-deletional mechanisms which limit T cell responses at the effector level. Finally, immune deviation or phenotypic skewing of the effector cells toward a non-pathogenic cytokine profile may also contribute to immune tolerance [reviewed in (54, 56–58)] (**Figure 1**).

The above mechanisms act on the autoreactive T cell itself to avoid its activation or dampen an already initiated immune response. T cell reactivity can also be indirectly modulated by other cell subsets with regulatory properties. Among these, regulatory T cells (Tregs) are a key component to maintain tolerance and towards active suppression of unwanted immune responses (**Figure 1**). Circulating regulatory T cells consist of different cell populations including naturally occurring CD4+ regulatory T cells (nTregs) (59) and type 1 regulatory T cells (Tr1) (60).

nTregs maintain self-tolerance and immune homeostasis. They are characterized by expression of the transcription factor Forkhead-Box-Protein P3 (FOXP3) and they are referred to as FOXP3+ Tregs. Most FOXP3+ Tregs arise in the thymus (thymus-derived Tregs or tTregs), but they can also be generated in the periphery (peripherally derived Tregs or pTregs) through conversion of conventional T cells (61, 62). During thymic selection, T cells recognizing self-antigens with intermediate avidity in between the range of positive and negative selection are not deleted and differentiate into tTregs (63). They enter the peripheral immune system in an already antigen-primed, activated, and functionally competent state. Additional markers of thymic/naive FOXP3+ Tregs like GPA33 have been identified (64), and the stability of these cells in the peripheral immune system is being investigated. Whether FOXP3+ Tregs need to be antigen-specific to exert their suppressive action remains to be clarified.

Tr1 cells are induced in the periphery after activation by specific antigen and characterized by secreting large amounts of IL-10, which constitutes their main suppressive effector function (65). Both FOXP3+ Tregs and Tr1 cells act through a range of mechanisms to regulate cells in close proximity and thereby can mediate bystander suppression against cells with different antigen specificities. For more detailed information on markers of Treg differentiation, function, tissue homing, and potential activation markers the reader is referred to reviews (62, 65, 66).

In summary, tolerance to self-antigens should be viewed as a complex and dynamic process resulting from several mechanisms acting in concert simultaneously or in a sequential manner to keep autoreactive cells under control (58). While mechanisms such as anergy or apoptosis are triggered at the initial phases after antigen encounter, regulatory T cells are presumed to contribute to long-term and stable tolerance induction and maintenance. Failure of these mechanisms leads to tolerance breakdown, enabling the development of autoimmune diseases.

## Tolerance-Inducing Approaches

A wide range of antigen-specific tolerization approaches have been tested in animal models of autoimmune diseases and particularly in EAE. The majority of these employed putative target antigens *via* different routes of administration, as peptides, proteins in free form, coupled to cells, MHC molecules or in the context of nanoparticles, *via* liposomes, or *via* expression vectors (11, 54, 67–69) and more recently as modified RNAs (70). Furthermore, they varied with respect to targeted organ and putative mechanism(s) of action, and their putative mechanisms of action, advantages and disadvantages have been reviewed elsewhere (11, 67, 68, 71). We have recently also reviewed those that have been tested already in MS (11) and will therefore focus on the principles here.

The ideal therapeutic approach for MS should aim to specifically silence the imbalanced self-directed immune responses by inducing long-lasting, stable immune tolerance against the target antigen (antigen-specific tolerization). Such an approach should leave immune effector functions, e.g. against infectious agents and tumors, intact and restore immune homeostasis. The ultimate goal is to deliver the autoantigen in a non-immunogenic context in order to exploit and enhance peripheral tolerance regulatory mechanisms and induce a durable state of immune tolerance (**Figure 1**). In the EAE model, antigen-coupled, cell-induced tolerance with myelin-derived antigens has consistently shown high efficacy both prophylactically and therapeutically and also prevented epitope spreading (72), but many other methods have been tested successfully as well (67, 68, 70–73). Although their mechanisms are not completely elucidated yet, free peptides or APLs are presumed to induce direct tolerance through anergy or phenotypic skewing (68), while tolerization by antigen-coupled cells or antigen-loaded nanoparticles involves more complex mechanisms. These include early PD-L1-mediated anergy of autoreactive cells, followed by induction of regulatory T cells for long-term tolerance maintenance, with IL-10 playing a key role in both processes (68, 73, 74).

In conclusion, major hurdles in translating these into patients include the differences of immune mechanisms between rodents and humans, e.g. with respect to the complexity of the MHC/HLA, the fact that EAE is an induced model, while MS arises spontaneously and probably long time before it becomes clinically manifest, the complexity and heterogeneity of disease mechanisms that contribute to MS, the still incomplete knowledge about target antigens, and, as will be detailed below, the difficulties of demonstrating tolerance induction and the underlying mechanisms in humans.

## Mechanistic Testing Along Clinical Trials Aiming at Tolerance Induction

For successful translation it is paramount not only to choose the target patient population, clinical trial design and outcomes well, but also to include a carefully designed mechanistic program that shows that antigen-specific immune tolerance has been achieved during the early clinical development stage. The form and stage of MS, ideally early RMS patients, who did not fail multiple prior treatments, the extent of disease activity, the reactivity against important target antigens and whether there is already epitope spreading or not, the HLA background, the presumed mechanisms, by which the respective tolerance-inducing strategy is supposed to work, and of course the safety of the approach, i.e. that it does not suppress the immune system or even lead to immune activation, all have to be considered. Most of the early tolerance trials in MS have not invested sufficient efforts to demonstrate that tolerance has been achieved at the mechanistic level. Furthermore, the high cost and the fact that a growing number of effective therapies are available have been reasons why such trials have been difficult to conduct. Below, we will address key mechanistic aspects to improve in this area in the future (for summary see also **Figure 1**).

### Immunosafety

The mechanistic studies performed along any tolerizing approach should first of all show that the treatment is not causing unwanted immune activation or signs of immunosuppression, i.e. that it is safe from an immunological point of view. Besides the standard hematology and blood chemistry analyses to check the general health status of the patients and identify potential adverse effects, flow cytometry is very useful to monitor immune cell composition after applying a tolerizing therapy. Current multicolor flow cytometry techniques allow the quick assessment of multiple parameters in parallel, thus enabling a comprehensive characterization of numerous immune cell subsets. Moreover, the use of immune cell profiling from fresh blood identifies potential changes in non- or minimally manipulated samples, thus getting a glimpse at the *in vivo* immunological status. Important aspects to take into account are the use of optimized and standardized methods of sample collection and processing, the timing of sample preparation, instrument settings, inclusion of counting beads and parallel measurement of absolute mononuclear blood cell numbers, in order to reduce variability between analyses as much as possible (75).

### Phenotypic Changes of Immune Cells

Several immune cell populations, including pro-inflammatory CD4+ Th1, Th1*, and Th17, memory B cells, CD8+ T cells, regulatory T- and B cells and others, are involved in the pathogenesis of MS. Disturbances in circulating immune cells have been reported in MS, and some of these alterations reflect those observed in the CNS (76). Detailed immune profiling of peripheral blood therefore not only provides information about the safety, but could also in principle be used to monitor changes related to disease activity and response to treatment.

In the context of tolerance induction the aim is to detect a shift in the pathogenic immune response from the Th1/Th1*/Th17 towards a “normal” one including the disappearance of pathogenic cells, appearance/activation of regulatory cell populations, or changes in markers indicative of tolerance. Multi-parametric flow cytometry techniques combining different surface markers (lineage markers, chemokine receptors, activation- and migration markers, antigen-induced T cell exhaustion and senescence markers) and intracellular staining to detect cytokines and transcription factors provide detailed information about phenotype, activation status, and functional profile of immune cells. Even more detailed analyses can be achieved with high resolution (up to 40 colors) flow cytometry, spectral cytometry (77, 78) and mass cytometry (CyTOF) (79, 80), but they are not used yet in the routine setting and, to our knowledge, have also not yet been employed in tolerance trials. Another recent development, the combination of oligonucleotide-bar-coded monoclonal antibodies against a wide range of surface markers of immune cells with single-cell RNAseq (referred to as Cellular Indexing of Transcriptomes and Epitopes by Sequencing; CITE-Seq), opens an entirely new level of information on immune cell composition, differentiation, functional phenotypes, and even TCR α/β expression. These methods are increasingly applied to characterize immune cell infiltrates in a variety of infectious-, inflammatory-, and autoimmune diseases and also in tumors (81–83). Since they have only recently been introduced and their bioinformatics analyses are very demanding, they have, to our knowledge, not been used in tolerance trials yet, but we find them very promising.

Despite these powerful analytical tools, the hypothetical and expected changes, which might be induced by a tolerization strategy, may still escape detection at the level of bulk PBMCs. Important reasons for this are the low precursor frequency of antigen-specific, proinflammatory T cells (see below) and the fact that MS patients are immunologically healthy, i.e. the disease-specific abnormalities are very subtle and do not lead to easily discernible alterations or general compromises of protective immune function.

Finally, a comprehensive mechanistic program should include the phenotyping and functional profiling of immune cells in the target organ, i.e. CNS-infiltrating cells. However, the low numbers of cells that can be obtained from CSF after a lumbar puncture, the obvious limitations for repeated spinal taps at different time points (compared to peripheral blood) and the fact that autoreactive T cells are expected to enter and leave the CSF and brain compartment, i.e. that they will not be there all the time, limits the usefulness, even if multiple spinal taps could be performed.

From a technical point of view, important aspects to consider are the use of fresh versus frozen material for the immune phenotyping, since sample processing and cryopreservation may impact the expression of several markers such as chemokine receptors or activation markers. Further, intracellular staining for cytokine detection requires the use of activators and fixation procedures, and cytokine production may be influenced by the activation method.

In summary, the comprehensive phenotypic characterization of immune cells before and after tolerization should at a minimum include multi-color flow cytometry panels to capture changes in the main immune cell populations and, in more detail, the phenotypes, migration markers and activation states of CD4+ T cells and B cells. We anticipate that the abovementioned novel techniques that combine surface markers with RNAseq will allow much more detailed analyses in the near future.

### Measuring Antigen-Specific T Cells

Documenting the effects of the respective tolerizing approach on the numbers and phenotype of autoreactive T cells is a prerequisite for demonstrating that it indeed induces antigen-specific tolerance. This aspect is currently one of the least well developed and most challenging. Below, we will cover important points that need to be considered.

There is solid evidence that certain immunodominant peptides of MBP, MOG, PLP and a few other non-myelin/non-CNS antigens appear involved in MS, and CD4+ T cells against are increased in MS, show higher antigen avidity, express proinflammatory phenotypes, and are frequently restricted by MS-associated HLA-DR molecules (4, 20–22, 26, 84). It is therefore important to document that a tolerizing approach either silences/anergizes these cells, deletes them or induces Tregs that control them. While this is obvious in theory, translating it into practice poses enormous challenges from several reasons including: a) the very low precursor frequency of autoreactive CD4+ T cells in the range of 10^-4^ - 10^-7^ depending on the assay (85–87), b) the methods that are available for reliably detecting these rare cells before tolerization or, even worse, their reduction after tolerization, remain poorly developed, c) demonstrating a change in phenotype of such rare cells in conjunction with their antigen specificity is also very difficult. Different from vaccination approaches, where one may start with a low precursor frequency T cell population, but wants to demonstrate its increase, the opposite, i.e. that very infrequent autoreactive T cells decrease or disappear, is a major challenge. Several methods are in principle available, and we list their main characteristics in **Table 1**. In short, they include detecting antigen-specific T cells by proliferative testing [3H-thymidine incorporation (88, 95); CFSE dilution (89)], ELISpot and FluoroSpot (90, 91), and FACS-based methodologies that measure the upregulation of CD154 or other surface markers after short-term, antigen-specific activation (92, 93), or using antigen-loaded HLA-class II tetramers (94). Besides the detection method, it is important to consider the organ compartment (peripheral blood lymphocytes versus CSF-infiltrating T cells), further the cell types (whole PBMC or memory T cells, freshly isolated versus frozen cells), the stimulating antigens (peptides versus whole proteins including controls to rule out effects on viral and bacterial recall antigens), and finally also to include antigens that might be important to capture effects on epitope spreading (29). After testing multiple different assay types and variables, we find the considerations that are summarized in **Table 2** helpful. May be with the exception of the FluoroSpot assay, which was recently introduced by Bronge et al. (90), none of these assay platforms is sufficiently well established and standardized to measure the frequency of autoantigen-specific T cells. Most require experience in cellular immunology techniques, and some should be performed preferentially with freshly isolated cells (96). HLA-DR/peptide tetramers have in our experience so far not worked at all for measuring autoreactive, peptide-specific T cells in bulk peripheral blood lymphocytes. The reasons probably include the low precursor frequency of autoreactive T cells, their too low antigen avidity, and the fact that only few specific HLA-DR/peptide tetramers are available. Recent developments enhancing tetramer binding and affinity may help to overcome these problems (97, 98), and combinations of tetramer staining and TCR sequencing to track antigen-specific T cells before and after tolerization could greatly improve it further.

**Table 1:** Assays for testing the frequency of autoantigen-specific T cells.

Assay methodology	Advantages	Disadvantages	Reference
(3H)-thymidine incorporation	Easy, high dynamic range, sensitive, easy to quantitate, well established, inexpensive	Requires radioactivity, takes several days, detected precursor frequencies low*	(88)
CFSE dilution	Easy, well established, allows characterization of the viability, phenotype and functional status by flow cytometry	Difficult to quantitate, insensitive, narrow, dynamic range, less data for use with autoantigens	(89)
ELISpot/FluoroSpot	Easy to use, relatively more reliable/standardized, detects frequency of cells with a specific functional phenotype (based on the detected cytokine/s), detects higher frequencies than proliferation, relatively robust	Overall less experience than with e.g. thymidine incorporation	(90, 91)
Upregulation of CD154	Fast (few hours), easy to quantitate, preferentially detects proinflammatory cells	Relatively insensitive, less data for use with autoantigens**, requires freshly isolated cells	(92, 93)
HLA-class II/peptide tetramers	In principle suited for direct detection of antigen-specific T cells, allows isolation of cells	Narrow dynamic range, insensitive, overall poorly developed for autoantigens***, few DR/peptide tetramer combinations available, promising in combination with TCR sequencing once available	(94)

*In the range of 10^-4^ to 10^-7^ for myelin-specific CD4+ T cells. Therefore, it is important to seed sufficient numbers of cells, i.e. minimally 5 and better 10 or more wells with 2x10^5^ cells/well. We have obtained better results with respect to background and number of positive wells with seeding CD45RA- cells, which contain memory T cells and monocytes/macrophages, but no B cells, which are often responsible for high background stimulation.

**Works best with freshly isolated cells; an assay that measures the upregulation of CD137 after antigen stimulus preferentially detects activated Tregs

***In our hands no HLA-DR/peptide tetramer from commercial- and academic sources has given reliable results so far; due to the fact that only few DR/peptide tetramers are available, even if they did work, one would have to use multiple ones to capture T cells restricted by all or most HLA-DR/DQ combinations that the patient expresses.

**Table 2:** Important considerations for measuring the frequency of autoantigen-specific CD4+ T cells.

1. Testing memory T cells (e.g. CD45RA- cells) is preferable over whole PBMCs
2. Seed sufficient numbers of cells/well and replicates in order to detect a meaningful number of autoreactive T cells before and after tolerance induction
3. Protein antigens that cover all potential epitopes are preferable to peptides
4. If peptides are used, focus on immunodominant epitopes and those peptides that are used in the respective tolerization approach
5. Stimulate cells with antigen at a low/intermediate concentration to increase the chance to measure high avidity T cells (e.g. 1-5 µM peptide)
6. Include peptides that are not part of the tolerization approach to detect epitope spreading and the influence of the tolerizing approach on these specificities
7. Include foreign antigen-derived peptides as controls, ideally peptides or proteins to which most humans react, e.g. tetanus toxoid, viral peptides from CMV, EBV, influenza (like CEFII)
8. Include sufficient numbers of cells/well and replicates that are not stimulated (negative control)
9. Define response criteria
10. Consider HLA-class II types of patients in the context of peptides in the tolerization approach and their known HLA-class II restriction of CD4+ autoreactive T cells

Defining clear response criteria, e.g. a 50% reduction of T cells with a given specificity, is important as is the timing, when responses are examined. Measurements at two time points prior to and also after tolerization are preferable, and, whenever frozen cells can be used, cells from time points before and after tolerization should be measured together to minimize inter-assay variations. Regarding the compartment from which the cells are derived, CSF appears preferable, since CSF-infiltrating T cells are likely important for the pathogenesis of the disease, however, several points need to be considered. Incorporating CSF testing and repeat spinal taps is rarely possible. Since the patient may refuse a second spinal tap, it carries the risk that no measurement after tolerization is available. Furthermore, the number of patients, who show positive responses of CSF CD4+ T cells to the abovementioned antigens, is in the range of 10-20% (99), probably because autoreactive T cells enter and leave the CSF compartment.

In summary, we currently favor the Fluorospot assay (90) or proliferation of CD45RA- memory T cells from the peripheral blood after pre-enrichment of these cells (14, 26, 88). It is currently open whether the rapidly improving single cell technologies, which can incorporate isolation of certain cell types or bar-coded antibody tagging and single cell RNA sequencing, or affinity-matured tetramers will resolve some of the above issues. While the single cell methods offer clear advantages such as information about the gene expression profile and TCR expression, their costs are still very high, bioinformatics are demanding, and, since only between 10.000-20.000 cells can be analyzed, their application for low frequency autoreactive T cells needs further refinements.

### Regulatory T Cells

The induction of stable regulatory cell populations that sustain a robust state of immune tolerance and over long periods of time or forever is an important goal of tolerizing approaches. Both FOX3-expressing Tregs and Tr1 are thought to be important in MS, although the data on circulating Tregs in MS and their involvement in its pathogenesis are conflicting. The paragraph below will summarize findings about Tregs with a focus on MS and how this information is relevant for antigen-specific tolerization.

Tregs are usually identified and their frequency assessed by flow cytometry. Their heterogeneity regarding phenotypes, differentiation and stability complicates Treg characterization before and after tolerization. Further, clear criteria for combinations of markers that unequivocally identify the different types of Tregs and address their functionality are still lacking.

FOXP3+ Tregs are defined as CD4+ CD25+ cells with low or no CD127 expression and expressing FOXP3. In MS, reduced (100), unaltered (101–103), but also increased (104, 105) numbers of Tregs have been reported. Other studies showed impairments of Treg function in MS patients (106, 107). These discrepancies are to some extent due to differences in the criteria for characterizing Tregs. Despite the archetypical high CD25 and low CD127 expression, there are multiple phenotypic subsets reflecting distinct differentiation- and activation states based on surface marker expression (like CD45 isoforms, CCR7 or HLA-DR) and with different suppressive capacities, which are, however, also not well characterized. FOXP3+ Tregs exert their suppressive effect by different mechanisms including IL-2 deprivation *via* CD25 capture, CTLA-4 expression, generation of immunosuppressive metabolites, release of immunosuppressive cytokines, and cytotoxic activity *via* perforin and granzyme, among others (62). Some of the surface markers used for their characterization are related to these mechanisms of action, yet none of them is specific for Tregs. CD39 is an ectoenzyme involved in ATP catabolism into cAMP and adenosine, which are immunosuppressive. CD39 identifies cells with an effector-memory phenotype that control Th17 responses, and CD39+ cells have been described to be decreased and functionally impaired in MS patients (108, 109), but increased levels have also been reported in RMS patients during relapses (110, 111).

FOXP3, the master regulator for the development and function of Tregs, remains their core marker (112, 113). Several studies have linked low FOXP3 expression levels with MS (100, 114), which were restored after vaccination with peptides of TCRs that are expressed by myelin-autoreactive cells (115). However, FOXP3 expression alone is not sufficient to identify Tregs, as the transcription factor is also transiently expressed in activated cells lacking suppressive ability (116). Stable FOXP3 expression in Tregs, and Treg lineage stability in general, is epigenetically regulated and relies on the presence of DNA demethylation in non-coding regions of the FOXP3 locus [Treg-specific demethylated region (TDRS)] and other Treg-associated genes such as IL2RA, CTLA4, IKZF2, and IKFZ4 (117–119). This epigenetic signature and not only FOXP3 expression marks *bona fide* Tregs and allows discrimination from activated CD4+ CD25+ FOXP3+ conventional T cells (120). Accordingly, it has been proposed that the term Treg should only be used for cells exhibiting the epigenetic Treg signature or with proven suppressive ability (61).

Therefore, the assessment of the methylation status of FOXP3 and the above genes could in principle give information about a favorable outcome of a tolerization strategy. However, these techniques are demanding and not established for routine enumeration of Tregs. In this regard, GPA33 (Glycoprotein A 33), a member of the immunoglobulin superfamily, has recently been reported to be a reliable marker of stable Tregs of thymic, but not peripheral origin, which show the epigenetic signature (64) and thus could be useful for the quantitative analysis of tTregs.

The identification of Tr1 cells is similarly complicated due to their low frequencies in peripheral blood, the lack of specific surface markers, and the inconsistent nomenclature in the literature. Like FOXP3+ Tregs, Tr1 cells display different suppressive mechanisms to regulate cells in their vicinity, such as IL-10 and TGF-β secretion, granzyme B- and perforin-mediated cytotoxicity, cell-contact dependent mechanisms or ATP catabolism. IL-10 is the Tr1 signature cytokine and critical both for their generation and function (65). Hence, Tr1 cells are best identified by their high IL-10 secretion. Functional defects of Tr1 cells have been described in MS patients, using IL-10 secretion *ex vivo* as indirect assessment of their suppressive function (121, 122), and during several tolerization trials in MS patients increased levels of Tr1 cells have been reported (123–125). However, the assumption that any IL-10-producing cell with suppressive capacity should be considered Tr1 is not correct (65), as other T cells can also release IL-10.

Gagliani and coworkers reported that the simultaneous expression of the surface markers CD49b and LAG-3 (Lymphocyte-Activation Gene 3) on memory CD4+ memory T cells (gated as CD3+ CD4+ CD45RA-) identifies Tr1 cells (126). This marker combination may not detect all Tr1 subsets, but only activated memory Tr1 cells. However, although some authors have argued that IL-10-producing CD4+ T cells constitute a heterogeneous cell population and that IL-10 is not an ideal marker for Tr1 cells (127), the current consensus is that CD4+ memory CD49b+ LAG3+ T cells, which produce high amounts of IL-10 and have regulatory activity independent from FOXP3, can be defined as CD4+ Tr1 cells (65).

The suppressive function of Tregs can be assessed with *in vitro* suppression assays (128, 129), which show the ability of a regulatory population to suppress the proliferation of conventional T cells in co-culture experiments. Suppression assays are relatively easy to perform, sensitive and inexpensive, and allow the parallel detection of cytokines in the supernatant. The use of CFSE to measure proliferation has advantages over the classical [H^3^]thymidine incorporation-based methods. It is not radioactive, more precise in assessing Treg function, since proliferation of Tregs cannot be excluded in [H^3^]thymidine incorporation assays, and shows the number of cell divisions and furthermore allows the phenotypic and functional characterization of the proliferating cells by flow cytometry (130). However, the *in vitro* conditions may not truly replicate the *in vivo* situation. Another drawback is the above-mentioned lack of well-defined surface markers for Tregs, which prevents the purification of homogeneous populations for the assays. Also, FOXP3 staining requires fixation of the cells which renders them useless for functional tests. Furthermore, is it not clear whether lack of suppression in MS is due to functional Treg defects and/or an increased resistance of autoreactive cells to suppression (44). Finally, due to the heterogeneous composition of both regulatory- and effector T cells, differences in their activation and state in the cell cycle, senescence and plasticity, these assays are very difficult to standardize, particularly if one uses bulk Treg and effector cell populations.

In summary, the identification of markers that are constitutively expressed by Tregs and reliably identify them as well as the development of a standardized functional assay would greatly help to assess Treg physiology and their role in MS, particularly in tolerization trials. It is possible that changes in the composition of Tregs rather than in the overall frequencies occur at different stages of the disease. In this context, FOXP3+ Tregs are believed to be essential for the initial phase of tolerance induction at the target organ, while Tr1 cells are key for the maintenance of long-term tolerance (65). Consequently, not only how they are assessed but also the time point of sampling after tolerization can influence the results of Treg analyses.

### Soluble Biomarkers of Inflammation and Reduced Target Organ Damage

We will briefly outline, which types of biomarkers exist and how they may aid mechanistic studies during tolerance induction.

According to standard pharmacology terminology, one can consider three types of biomarkers. a) Pharmacodynamic markers that are directly related to the mechanism of action of the tolerization approach. The induction of Tregs and their stability (e.g. TSDR demethylation), reduction of autoreactive T cells and respectively the induction of regulatory- (e.g. IL-10) or decrease of proinflammatory cytokines (IFN-γ, IL-17, GM-CSF) fall in this category. b) Pharmacokinetic markers, which allow determining the onset and duration of the effects. The same set of markers could be applied here. The fact that both proinflammatory and anti-inflammatory cytokines are found in serum or plasma at low picogram levels poses another hurdle to demonstrate differences from before to after tolerization. For conventional ELISAs these values are at the detection limits, and they are thus usually not suited. However, newer, more sensitive techniques as for example the single molecule array (SIMOA) methodology, or electrochemiluminescence-based assays, allow the reliable detection of cytokines even at these values. c) Finally, there are biomarkers that serve as an indirect readout for reduced target organ damage or inflammation, but are not specific for tolerization. As markers of target organ damage, i.e. damage of neurons and axons, and indirectly also an indicator of reduced tissue inflammation in the brain, neurofilament light chain (NfL), that can be measured in serum or plasma and CSF, is probably the best examined and validated (for serum) biomarker (131, 132). Chitinase 3-like protein 1 (CH3L1) reflects innate immune activation and inflammation (133), but is relatively unspecific. Other analytes reflect different aspects of the pathomechanisms of MS such as de- and remyelination (myelin proteins, oligodendrocyte differentiation markers), microglia and astroglia activation (e.g. GFAP), metabolic changes, and adaptive immune cell infiltration/activation. For detailed discussion, the reader is referred to reviews of biomarkers in MS (132, 134, 135). Since protein-measuring methodologies are robust and likely more relevant than changes in gene expression, which can be detected by multiplex quantitative PCR or RNAseq methodologies, they should be included in the mechanistic program. During early stage trials, a broader panel that is less hypothesis-driven and instead discovery-oriented is of interest. Several platforms offer the measurement of large(r) panels (up to 100 and more) analytes including mesoscale discovery, flow cytometry, bead-based methods, OLINK, SIMOA, and others. Some offer preset collections of analytes that are known to be related to inflammatory-, neurological-, or autoimmune conditions and can be analyzed in very small sample volumes. Performing genome-wide RNAseq with cells of interest, e.g. CD4+ T cells or B cells, at the bulk or single cell level affords an even broader look with the caveat that increased gene expression not necessarily translates into increased protein expression and that quantitation remains more difficult. The latter methodologies can be combined with stimulation by global- or antigen-specific activation, but such steps have so far not been applied in tolerance approaches. Further, a number of proteomics technologies that measure either large sets of defined markers (e.g. SOMAscan; SomaLogic) or even broader sets (e.g. SWATH-MS; Creative Proteomics), that aim to overcome one core issue, i.e. the very low concentrations of immunologically relevant analytes in serum/plasma, have been developed and are beginning to be tested. Similar to the above single cell RNAseq methodologies, proteomics- and also epigenetic profiling methods will likely not only be applied alone, but also in combination in the future.

In summary, soluble analytes that can serve as pharmacodynamic and pharmacokinetic measures are currently very scarce. Biomarkers of target damage or -inflammation are not specific for tolerance induction and probably only change with some delay. Despite these problems carefully collected, processed and cryopreserved samples from tolerization trials are not only an invaluable resource for exploratory studies that are already feasible, but furthermore can be used in the future, if improved methods become available.

### Other Important Aspects

Dose finding: Mechanistic studies along tolerance-inducing therapies (for a summary of suitable assays see **Table 3**) can assist in identifying the best dose of the respective regimen. In some previously tested approaches in animal models, the dose range appeared critical for achieving the desired effect, and in MS trials with different tolerizing therapies such as oral tolerance (136), altered peptide ligand vaccination (95, 137), and also subcutaneous administration of peptides (124), there was no linear relationship between dose and effect, but rather a critical/optimal or even damaging dose range. Tolerization with peptide-coupled splenocytes in EAE and other animal models of autoimmune diseases showed a threshold dose, below which no effect was observed (138), but an upper limit had not been seen with the relatively narrow span of doses that had been tested. There is no well-established formula that would allow to extrapolate dose ranges from animal testing to humans in the field of tolerance induction. One should therefore try to gather as much information as possible from mechanistic studies in parallel to surrogate outcomes such as magnetic resonance imaging lesions during early clinical testing. In addition to capturing tolerance-related effects, it is also imperative throughout the clinical development program to assure that the respective, supposedly tolerance-inducing therapy is safe and does not induce rather than attenuate proinflammatory autoimmune reaction. The latter has been observed with the highest dose of an altered peptide ligand of MBP 83-99 (95) underscoring that the documentation of immunosafety is a key goal.

**Table 3:** Important components of a mechanistic studies/biomarker program to test immune tolerance in multiple sclerosis.

Goal	What should be measured/method	Comments
Immunosafety	Increase of autoreactive T cells	Multiple time points and at least two complementary methods
	Change to more proinflammatory phenotype	
	Exclusion of immunosuppression and major alterations of immune cell composition	
Reduction of autoreactive T cells	Decrease of autoreactive T cells	For details, see **Tables 2**, **3**
	ELISpot/FluoroSpot with whole antigen	
	Proliferation assay with peptides	
Effects on FOXP3+ Tregs, Tr1 cells	Flow cytometry protocols using several markers	Consider epigenetic modifications of TSDR*
	Suppressive function of Tregs	
Pharmacodynamic soluble biomarkers	IL-10	Use highly sensitive assay; made by multiple cell types besides Tr1 cells
Biomarkers for tissue damage	Neurofilament light chain	Use highly sensitive assay
Biomarkers for inflammation	Chitinase 3-like protein 1, others	As above
Exploration of previously unknown mechanisms and cell types	RNAseq in single cells, ideally in combination with methods that allow measuring transcription in defined cells (e.g. by bar-coded antibodies against immune cell surface markers)	Data analysis still challenging; several methods in development
	Proteomics techniques suited to measure large numbers of analytes	Several methods and approaches; technically demanding to measure low-abundance molecules in serum/plasma

*TSDR, Treg-specific demethylated region.

Duration of tolerization effects: Mechanistic readouts, for example the reduction of autoreactive T cells or the induction of different types of Tregs, can also help in determining how long a tolerizing effect lasts and when retreatment may be needed (see above for pharmacokinetic markers). A pertinent example is again tolerization with peptide-coupled splenocytes in EAE (68), which is not only very effective when applied prophylactically and therapeutically, but also shows remarkably long-lasting effects. A single tolerization is usually sufficient in autoimmune models to protect the animal lifelong from re-induction of the autoimmune disease (68). In humans, we do not know if tolerization effects, if they can be induced, will last equally long, but assume that periodic re-treatment will be necessary even with peptide-coupled cell-based tolerization. Accordingly, periodic testing of the putative tolerizing effects should be incorporated particularly during phase II testing.

Patient selection: Autoreactive CD4+ T cells recognize peptides in the context of specific HLA-class II molecules, for example MBP 111-129 together with HLA-DRB1*04:01 (139), or GDP L-fucose synthase with DRB3*02:02/03:01 (13, 99) while others, for example MBP 83-99 is a promiscuous HLA-class II binder and immunodominant not only in the context of the DR15 alleles DR2a and DR2b, but also other DR alleles (140). Assuring that the patient population of a tolerance trial is representative with respect to HLA-class II types in the context of the tolerizing antigens is therefore important. Futhermore, patients at early stages of CIS or RMS with inflammatory disease activity as measured by MRI are probably the ideal group for early-phase tolerization trials and most informative. Patient selection should therefore consider inclusion of the most prevalent, MS-associated HLA-class II alleles, robust reactivity to at least one of the tolerizing autoantigens to be able to measure changes, and patients with early active MS rather than in the progressive stage and after failing multiple MS drugs before. For further details see (11).

## Conclusions and Outlook

Incomplete understanding of peripheral immune tolerance and how specific tolerance approaches work in humans, the frequent omission to include mechanistic studies along early clinical trials and also the lack of reliable methods to measure for example the reduction of autoantigen-specific T cells or the induction of Tregs are possible reasons why prior efforts failed. This short review emphasizes the importance of mechanistic- and biomarker studies for the clinical development of tolerance-inducing approaches. These should be tailored to the respective approach and its putative mechanism(s) of action. Carefully developed standard operating procedures and validation of different methods are necessary. Similar to the use of imaging parameters, which have been accepted as surrogates for clinical efficacy in phase II clinical trials in MS, a core set of mechanistic studies and biomarkers should be incorporated. This should at least include measuring the reduction of antigen-specific T cells, the changes of natural- and induced Tregs and pharmacodynamic biomarkers such as IL-10, but also markers depicting damage of the target tissue as for example NfL. Further, it is desirable that these are measured in a standardized fashion across clinical trials and different approaches to reach a consensus on methods and analyses, which in turn should help in understanding and comparing immunologic effects of therapeutic approaches and support clinical development and interaction with regulators. In the US, the Immune Tolerance Network (ITN), which exists for more than a decade and is jointly sponsored by the National Institutes of Health and the Juvenile Diabetes Research Foundation, has invested a lot of effort in developing standardized assay protocols, pursued some of the above aspects already along trials, e.g. in type I diabetes (54), and also sponsored tolerization trials (https://www.immunetolerance.org/). Their efforts have been instrumental in systematically addressing several of the challenges that we mention, but since the pathogenic mechanisms differ between diseases as well as the knowledge on target antigens and tolerance mechanisms, it would be highly desirable to intensify international exchange and collaborations further in specific disease areas in the future to harmonize mechanistic studies along tolerization trials.

## Author Contributions

All authors declare that they have substantially participated in the preparation and writing of the manuscript and have taken due care regarding their contribution to ensure the integrity of the work. All authors contributed to the article and approved the submitted version.

## Funding

The development of peptide-coupled cell tolerance in MS, which provided a frame for the above, was supported by the Wyss Zurich (ETIMS^red^ project), by Swiss National Science Foundation (SNF) grant (32003B_185003) to RM, and further by the Clinical Research Priority Projects (Heterogeneity of MS; Precision-MS) of the University of Zurich.

## Conflict of Interest

RM received unrestricted grant support from Biogen, Novartis, Hoffman La Roche and Third Rock, and compensation for advice or lecturing by Biogen, Novartis, Sanofi Genzyme, Merck, Hoffmann La Roche, Neuway, CellProtect, and Abata. RM is employed part-time by Cellerys, a startup-company outfounded from the University of Zurich. He is a co-founder and stockholder of Cellerys, and a co-founder of Abata Therapeutics. AL received financial compensation and/or travel support for lectures and advice from Biogen, Merck, Novartis, Teva, Genzyme, Bayer and Celgene. AL is employed by Cellerys. He is a co-founder and co-owner of Cellerys. MS is a co-founder and co-owner of Cellerys. RM, MS, and AL are listed as inventors on patents of the University of Zurich about target antigens in multiple sclerosis. RM is further listed as inventor and received remuneration for a NIH-held patent on the use of daclizumab to treat multiple sclerosis.

The remaining author declares that the research was conducted in the absence of any commercial or financial relationships that could be construed as a potential conflict of interest.

## Publisher’s Note

All claims expressed in this article are solely those of the authors and do not necessarily represent those of their affiliated organizations, or those of the publisher, the editors and the reviewers. Any product that may be evaluated in this article, or claim that may be made by its manufacturer, is not guaranteed or endorsed by the publisher.
